# Effective Multi-Mode Grasping Assistance Control of a Soft Hand Exoskeleton Using Force Myography

**DOI:** 10.3389/frobt.2020.567491

**Published:** 2020-11-16

**Authors:** Muhammad Raza Ul Islam, Shaoping Bai

**Affiliations:** Department of Materials and Production, Aalborg University, Aalborg, Denmark

**Keywords:** human intention detection, FSR sensor band, exoskeleton control, grasping assistance, soft hand exoskeletons

## Abstract

Human intention detection is fundamental to the control of robotic devices in order to assist humans according to their needs. This paper presents a novel approach for detecting hand motion intention, i.e., rest, open, close, and grasp, and grasping force estimation using force myography (FMG). The output is further used to control a soft hand exoskeleton called an SEM Glove. In this method, two sensor bands constructed using force sensing resistor (FSR) sensors are utilized to detect hand motion states and muscle activities. Upon placing both bands on an arm, the sensors can measure normal forces caused by muscle contraction/relaxation. Afterwards, the sensor data is processed, and hand motions are identified through a threshold-based classification method. The developed method has been tested on human subjects for object-grasping tasks. The results show that the developed method can detect hand motions accurately and to provide assistance w.r.t to the task requirement.

## 1. Introduction

Grasping tasks are performed repeatedly in both the home and in workplaces. Studies have shown that a human in a work/home environment performs grasp and transition between different grasps approximately 4,700 times within a 7.45 h window (Zheng et al., 2011; Bullock et al., 2013). Performing these tasks repeatedly over a longer period of time can cause fatigue and injuries. Hand exoskeletons (Gull et al., 2020) have the capability to assist in these tasks, which can reduce human effort and the risk of getting injured/fatigued.

Proper control of the exoskeleton depends mainly on accurate human intention detection. Several methods to determine human intention that are based on electromyography (EMG) (Anam et al., 2017; Meng et al., 2017; Pinzón-Arenas et al., 2019; Qi et al., 2019; Zhang et al., 2019; Asif et al., 2020) and force myography (FMG) (Islam and Bai, 2019; Xiao and Menon, 2019, 2020) have been proposed. Leonardis et al. (2015) used EMG to control a hand exoskeleton for bilateral rehabilitation. Here, a paretic hand was provided with grasping assistance by estimating the grasping force of the non-paretic hand. In another work (Lu et al., 2019), pattern-recognition-based hand exoskeleton control was proposed for spinal cord injury patients. An FMG-based hand gesture classification method was proposed to control a hand prosthetic device in Cho et al. (2016). In total, 10 hand grips were classified using a linear discriminant analysis technique. A high-density force myography-based hand and wrist gesture classification approach was proposed by Radmand et al. (2016). It was shown that for static hand postures 0.33% RMSE is achieved. While variation in upper limb position reduces the accuracy, better performance can be achieved by introducing limb position variation in training protocol. Several other works on force estimation and pattern-recognition-based hand exoskeleton control have also been reported (Wege and Zimmermann, 2007; Rasouli et al., 2016; Ferigo et al., 2017; Secciani et al., 2019; Arteaga et al., 2020).

In all of the reported works, methods to control a hand exoskeleton are limited to either pattern recognition or force estimation. Furthermore, in these methods machine learning and deep leaning techniques are used that requires large training datasets to achieve good classification/estimation accuracy.

In this work we develop a new sensing method for both pattern recognition and force estimation using FMG. A multi-mode task detection approach, i.e., motion classification and grasp force estimation, is proposed for controlling a hand exoskeleton. In this method, four hand motion states are classified i.e., rest, open, close, and grasp. The classification algorithm is based on threshold approach and requires a small training dataset. Once the grasp is detected, the control mode is switched to grasp assistance. This is achieved by virtue of two sensor bands built with FSRs, which can detect muscle activities conveniently and effectively. In terms of its sensing method, FMG has exhibited a better performance than EMG in classification and estimation tasks considering accuracy, repeatability, and cost (Ravindra and Castellini, 2014; Jiang et al., 2017). Moreover, unlike EMG, FMG is not affected by skin conditions and has a simple electronics interface.

This paper is organized as follows. The design and implementation of the sensor band and exoskeleton control strategy are described in section 2. Section 3 presents the data processing and algorithm design for grasp detection and assistance. Experimental setup and testing results are described in section 4. Discussion on the developed method is presented in section 5. The work is concluded in section 6.

## 2. Materials and Methods

In this section, a methodology to detect hand motions i.e., rest, open, close, and grasp is described. Sensor bands, a hand exoskeleton, and control methods are also presented.

### 2.1. Methodology

In this work, four hand motion states are classified, i.e., rest, open, close, and grasp. The last three motion states are classified as dynamic states, whereas rest is identified as a static hand state in any posture, e.g., keeping the hand fully opened/closed or holding an object in a fixed posture.

In object grasping, fingers have to be flexed. During flexion, the muscle belly shortens in length and contracts toward the side of the elbow joint, which is referred as isotonic muscle contraction. As the object comes into contact with the hand, muscle shortening stops, and an isometric contraction state is initiated. In this state the muscle belly along the forearm contracts as long as the force applied to hold an object reaches the steady state.

In this work contraction states and the transition between them, i.e., isotonic and isometric, are measured through FMG, using sensor bands built with FSR sensors. In this method, normal forces caused by muscle contraction and applied to the sensor band, hereafter called muscle contraction-induced (MCI) force, are measured. Flexor digitorum profundus and flexor digitorum superficialis are the prime muscles that govern fingers flexion to close the hand. During hand closing movement, the length of these muscle shortens and they contract toward elbow joint. MCI force near the elbow will therefore increase, while it will decrease near the wrist joint. As soon as the object is grasped, muscles stop shortening and isometric contraction takes over. In this case, MCI forces over the muscle belly will increase. This principle can be expanded further to explain hand opening task. In hand opening the object is ungrasped, MCI force on both ends of the forearm will decrease. On the other hand, as the object is released and the fingers are further extended, MCI force measured near the elbow will decrease, while the force measured near wrist will increase. From these changes of MCI force, hand motion states can be determined with certain algorithms.

### 2.2. Sensor Band

**Figure 1:**
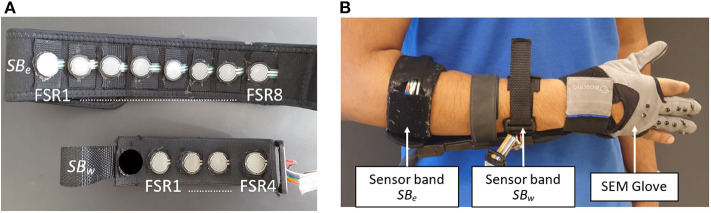
**(A)** FSR sensors placement inside sensor bands *SB*_*w*_ and *SB*_*e*_ and **(B)** SEM Glove and sensor bands placement on forearm.

The aforementioned hand motion detection relies on an effective and convenient method to detect MCI forces. To this end, two sensor bands are constructed at Aalborg University exoskeleton lab, as shown in Figure 1A.

The sensor bands are designed to be placed on the forearm, as shown in Figure 1B. One is placed near the elbow joint. This band, referred to as *SB*_*e*_, is comprised of eight FSR sensors. The other band is placed near wrist joint, referred to as *SB*_*w*_, which has an array of four FSR sensors embedded. The placements of FSR sensors inside the sensor bands are shown in Figure 1A. All FSR sensors are FSR-402, developed by Interlink electronics, and have the capability of measuring 0.1–10 N. More information on the construction of sensor bands can be found in Islam and Bai (2019).

### 2.3. SEM Glove

In this work a soft hand exoskeleton SEM Glove (Nilsson et al., 2012; Hashida et al., 2019) is used to provide physical grasping assistance, as shown in Figure 1B. The SEM Glove is equipped with FSR sensors placed at the middle and index fingertips and at the thumb. The assistance provided by the exoskeleton can be measured by these sensors. Moreover, in the SEM Glove's own control unit, the assistance level is also controlled using the same sensor data. The tighter the object is grasped the higher the assistance level will be. In this work, the assistance level provided by SEM Glove is controlled through MCI force measured by the sensor band placed near elbow joint instead of using the SEM Glove's own sensors.

### 2.4. Sensing Data

**Figure 2:**
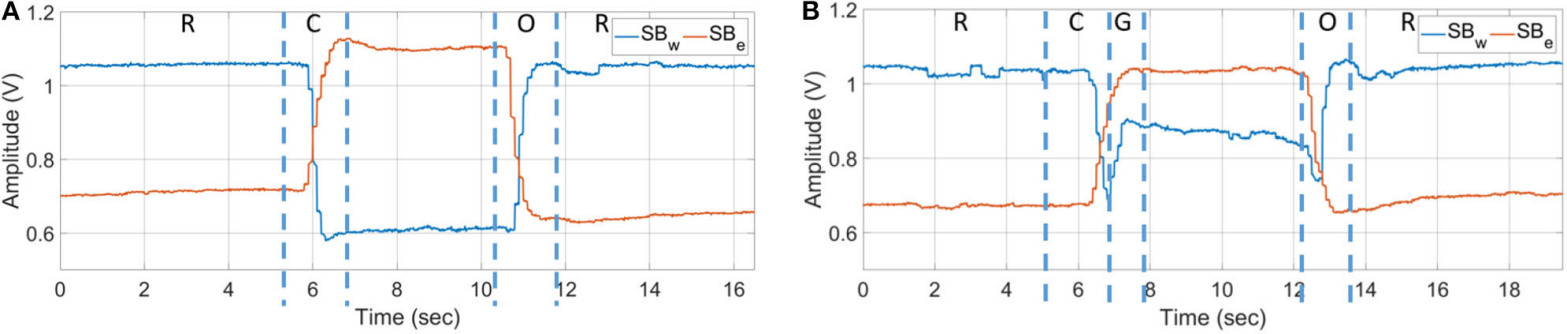
Net output voltage measured from sensor bands for opening and closing of hand **(A)** without grasping and **(B)** grasping an object.

The sensor bands allow us to collect hand motion data effectively. An example of a dataset of rest, open, close, and grasp, labeled as “R,” “O,” “C,” and “G,” respectively, is shown in Figure 2. Isotonic contraction during opening and closing of hand can be seen in Figure 2A. Figure 2B shows the data of an object being grasped when isometric contraction occurs. The state when the object is grasped is labeled as “G.”

In the hand opening task, shown in Figure 2B, it can be seen the sensor amplitude first goes down. This muscle activity represents loosening of grip on the object. Afterwards, increase in muscle activity at *SB*_*w*_ and decrease in muscle activity at *SB*_*e*_ are observed, which represents fingers extension to open the hand. In the implementation phase, loosening of grip is treated as a steady state.

### 2.5. Multi-Mode Control

**Figure 3:**
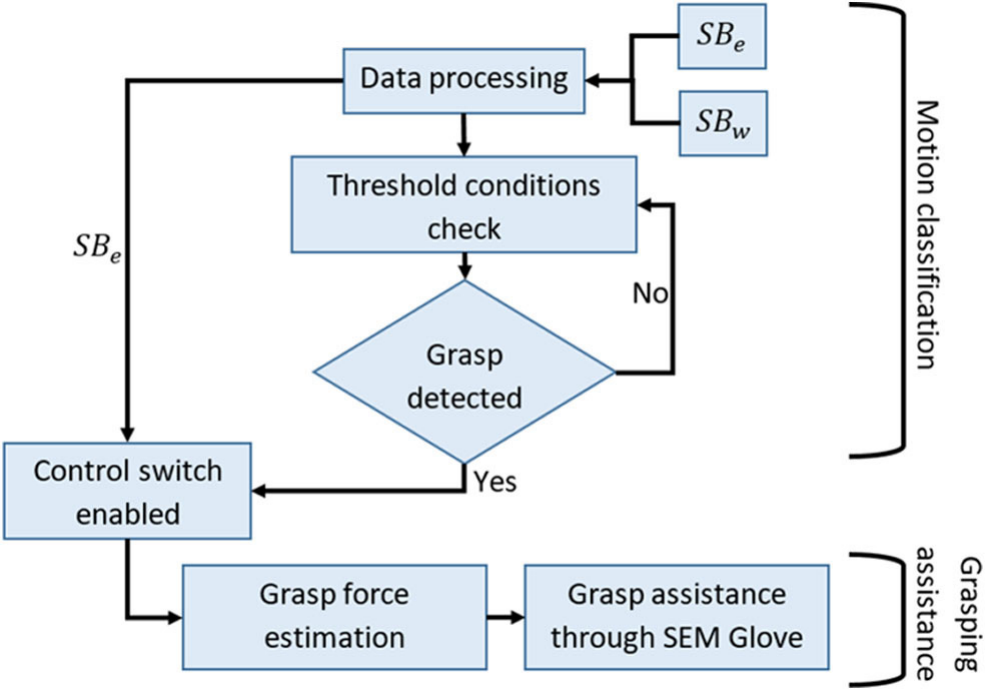
Flow chart of multi-mode control method.

In this work, a multi-mode control approach is used to assist in grasping, which is shown in Figure 3.

The control strategy is divided into two stages i.e., motion classification and grasp force assistance. Motion classification is based on a threshold approach. Out of four actions, i.e., rest, open, close, and grasp, once the algorithm identifies grasp action, the control mode is shifted to grasp force assistance. In this mode a proportional control is implemented, where the assistance force is determined using MCI force measured through *SB*_*e*_.

## 3. Data Processing

### 3.1. Sensor Calibration

The FSR sensors in the two sensor bands are interfaced with a non-inverting amplifier. The output voltage of the amplifier is thus given by the following equation:

(1) $$\begin{array}{l} V_{out}=\left(1+\frac{R_{ref}}{R_{fsr}}\right)V_{in} \end{array}$$

Here, *V*_*out*_ is the output voltage of the amplifier, *V*_*in*_ is the input voltage applied to positive terminal of the amplifier, *R*_*ref*_ is the reference resistance, and *R*_*fsr*_ represents the FSR resistance, which varies with force applied on it.

With the amplifier designed, it is possible to change the range of force measured by FSR. This is done either by changing the reference resistance *R*_*ref*_ or input voltage *V*_*in*_. In our design, the reference resistance is fixed to 100 *kohm*. We therefore adjust the input voltage *V*_*in*_ through a DAC port from micro-controller for this purpose, which is a task of sensor calibration.

In the calibration stage, input voltage *V*_*in*_ is adjusted so that at least three of the FSR sensors from both *SB*_*e*_ and *SB*_*w*_ have reached the maximum voltage limit. In this way, the sensor bands can have high resolution in all detections.

**Figure 4:**
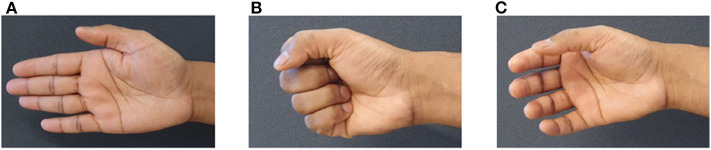
Gestures used in calibration and training stage. **(A)** open hand gesture to calibrate *SB*_*w*_, **(B)** close hand gesture to calibrate *SB*_*e*_, and **(C)** rest state gesture to collect data for threshold determination.

During calibration of *SB*_*w*_, the subject is asked to keep the hand open, as shown in Figure 4A. This posture initiates the calibration procedure. An automated program checks the sensors outputs above threshold level. If the number is less than three, input voltage *V*_*in*_ is increased gradually until the condition is fulfilled, i.e., at least three sensors are above threshold limit. Similar procedure is followed for the calibration of *SB*_*e*_ but for the close hand gesture, as shown in Figure 4B, to complete the calibration. In the current setup it is set to 1.5 V.

**Figure 5:**
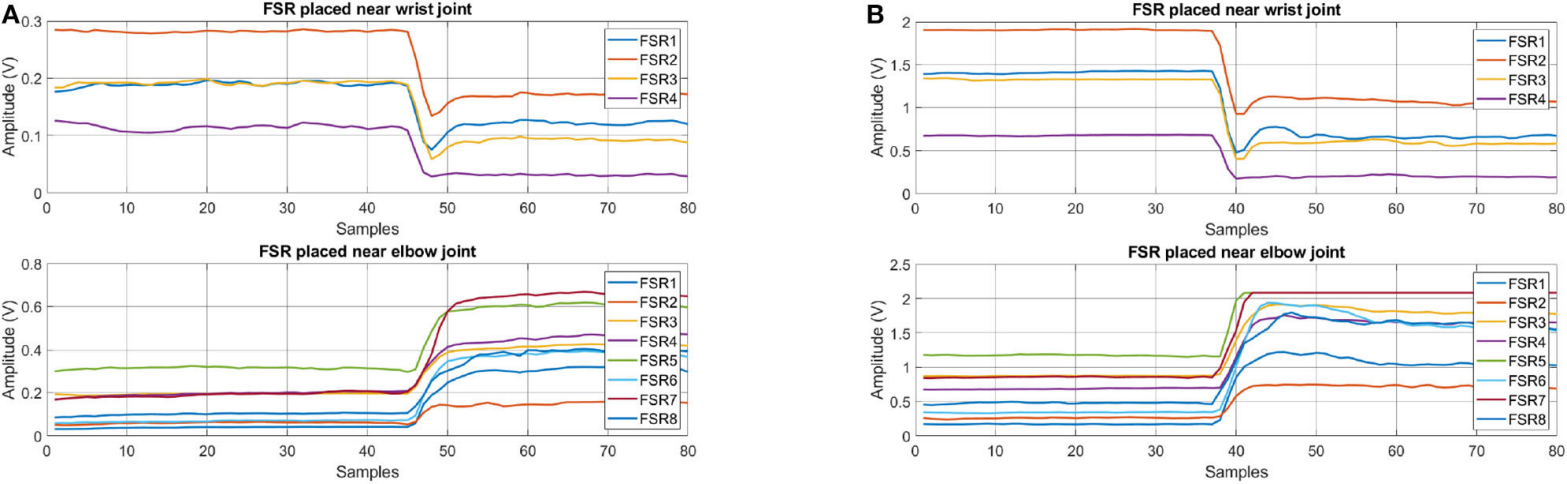
FSR data for hand closing gesture **(A)** before and **(B)** after calibration.

An example dataset of the calibration stage is shown in Figure 5. This dataset represents the task of hand closing from fully opened state. Figure 5A is the dataset collected before calibration and Figure 5B is the dataset collected after calibration.

The improvement in signal resolution, ν, is computed by taking the ratio of change in signal amplitude, from open to close hand gesture, to the standard deviation of signal value during the steady state condition. Mathematically it is given as,

(2) $$\begin{array}{l} \nu =\frac{\left|\mu \left(V_{O}\right)-\mu \left(V_{C}\right)\right|}{\max\left(\sigma \left(V_{O}\right),\sigma \left(V_{C}\right)\right)} \end{array}$$

Here, *V*_*O*_ and *V*_*C*_ represent the net voltage measured from the sensor bands for open hand and close hand gestures respectively, and μ and σ are the mean and standard deviation respectively. The results obtained through aforementioned equation are provided in Table 1. The results clearly show that the resolution of both sensor bands is increased significantly, more than two times, after calibration.

**Table 1:** Resolution measured before and after calibration.

**Sensor band**	**Resolution ν**		**% increase**
	**Without calibration**	**With calibration**	
*SB*_*w*_	27.88	60.13	221
*SB*_*e*_	27.94	61.74	222

### 3.2. Features Selection

While grasping an object, sensor readings highly depend on the shape and weight of the object. Moreover, donning and doffing of the sensor band also affects the sensor response. Developing a threshold- or machine-learning-based task-detection algorithm will require a large amount of data if the signal amplitude or it's RMS value is used as the input feature. It is noted that when a user takes off the sensor band and puts it back on, it is desirable that the sensor band has to be placed exactly at the same place and with the same tightness, but this is very challenging. All these factors will affect the classification performance.

**Figure 6:**
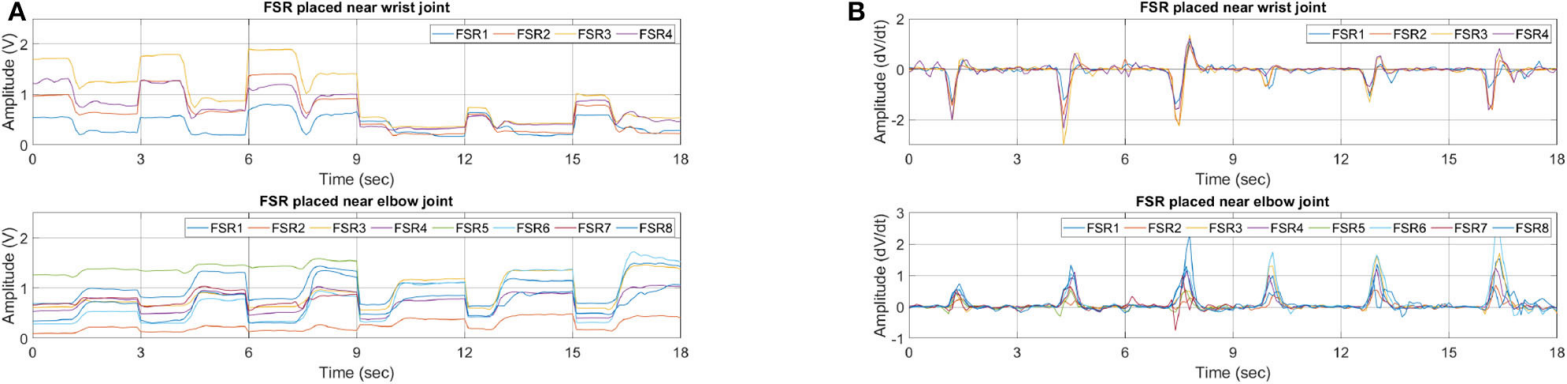
FSR feature dataset for grasping objects of different sizes and weights. **(A)** RMS and **(B)** slope.

With experiments, it is observed that the feature that gives consistent results with less deviation is slope. This feature represents the change in signal amplitude w.r.t time. An example dataset of grasping different shape and weight objects is shown in Figure 6. A grasping dataset for each object is represented in 3s windows. From time 0 to 3, 3 to 6, and 6 to 9 s objects A, B, and C are grasped sequentially, as shown in **Figure 8**. From 9 to 18 s a dumbbell bar is grasped three times with different weight hanged on the sides of it. The weights of the dumbbells, applied from *t* = 9 to 12, 12 to 15, and 15 to 18 s were 1.2, 2.3, and 3.4 kg, respectively. Data sessions from 0 to 9 and from 9 to 18 s were recorded separately. It can be seen from Figure 6A that there is big variation in FSR reading, as it depends on the shape and weight of the object. However, if we look at the slope feature in Figure 6B, a similar pattern but with different peaks can be observed. Initially, fingers are flexed therefore we see opposite slopes for the FSR sensors placed near elbow and wrist joint. As soon as an object is grasped, positive slopes for both sensor bands are observed. By carefully selecting the threshold value, grasp action can be detected very effectively. In this work we therefore selected slope feature for detection of hand motion.

### 3.3. Features Extraction

Two features are extracted from raw sensor data, i.e., root mean square (RMS) and slopes. RMS from raw sensor data is obtained using a 150 ms window in which 100 ms is non-overlapping and 50 ms is overlapping from previous window. After calculating RMS values for each FSR sensor, slopes are obtained using the following equation:

(3) $$\begin{array}{l} \kappa =\frac{R^{i}-R^{i-1}}{t_{ws}} \end{array}$$

Here, κ represents the slope feature, *R*^*i*^ represents the newest sample of RMS data, and *t*_*ws*_ is the window time to extract features.

### 3.4. Threshold Determination

In this method each state, i.e., rest, open, close, and grasp, is identified using a threshold-based classification approach. To determine the threshold limits, subject is asked to hold rest state, as shown in Figure 4C, for 5 s. Raw data obtained in this task is post processed to obtain slopes, which are further used to determine threshold limits.

After the computation of slope feature, the minimum and maximum slope value for each FSR was computed:

(4) $$\begin{array}{l} \xi_{w}^{max}=max\left(\Delta_{w}\right),\;\xi_{w}^{min}=min\left(\Delta_{w}\right) \end{array}$$

(5) $$\begin{array}{l} \xi_{e}^{max}=max\left(\Delta_{e}\right),\;\xi_{e}^{min}=min\left(\Delta_{e}\right) \end{array}$$

with

(6) $$\begin{array}{l} \Delta_{w}=\left[\kappa_{w}^{1}\;...\;\kappa_{w}^{N}\right],\;\Delta_{e}=\left[\kappa_{e}^{1}\;...\;\kappa_{e}^{M}\right] \end{array}$$

Here, *N* and *M* are the numbers of FSR sensors embedded inside the sensor bands *SB*_*w*_ and *SB*_*e*_, respectively. $\xi_{w}^{min}$ and $\xi_{w}^{max}$ are row matrices of order 1 × *N* and contain minimum and maximum slope values of *SB*_*w*_ sensor band data computed for rest state. $\xi_{e}^{min}$ and $\xi_{e}^{max}$ are also row matrices of order 1 × *M* and contain minimum and maximum slope values of *SB*_*e*_ sensor band data. Δ_*w*_ is a *I*×*N* matrix, where *I* is the number of slope feature samples computed from rest gesture data, and Δ_*e*_ is also a matrix but of *I*×*M* dimension.

Using (4) and (5), threshold conditions to detect each task are given as

(7) $$\begin{array}{l} H_{R}=\Delta_{w}^{r}<=k\xi_{w}^{max}\;\&\;\Delta_{e}^{r}<=k\xi_{e}^{max} \end{array}$$

(8) $$\begin{array}{l} H_{O}=\Delta_{w}^{r}>k\xi_{w}^{max}\;\&\;\Delta_{e}^{r}<k\xi_{e}^{min} \end{array}$$

(9) $$\begin{array}{l} H_{C}=\Delta_{w}^{r}<k\xi_{w}^{min}\;\&\;\Delta_{e}^{r}>k\xi_{e}^{max} \end{array}$$

(10) $$\begin{array}{l} H_{G}=\Delta_{w}^{r}>k\xi_{w}^{max}\;\&\;\Delta_{e}^{r}>k\xi_{e}^{max} \end{array}$$

Here, *H*_*R*_, *H*_*O*_, *H*_*C*_, and *H*_*G*_ are the thresholds for rest, open, close, and grasp task detection. $\Delta_{w}^{r}$ and $\Delta_{e}^{r}$ are row matrices that are computed during real-time testing. The information in these matrices is in same order as in Δ_*w*_ and Δ_*e*_.

### 3.5. Grasp Force Estimation

During the motion classification stage, if grasp action is detected, the control method is switched to grasp assistance. In this mode, we need to determine and control the grasp assistance provided by the SEM Glove. In this work, it is determined using the following equation:

(11) $$\begin{array}{l} u=\left(SB_{e}^{rms}-LB_{e}\right)K \end{array}$$

Here, *u* is the control input relayed to the SEM Glove, *K* is the proportional gain and $SB_{e}^{rms}$ is the net FSR output measured from the sensor band *SB*_*e*_. *LB*_*e*_ is the net FSR output measured at the time of grasp detection and is given by following equation:

(12) $$\begin{array}{l} LB_{e}=mean\left(R_{e}^{i},R_{e}^{i-1}\right) \end{array}$$

Here, *i* is the sample when grasp action was detected, and *i*−1 represents the sample before.

### 3.6. Performance Analysis

The performance of the task detection technique is analyzed with a group of four parameters, namely, precision, recall, F1-score, and accuracy (Powers, 2011). Mathematically, these parameters are calculated by

(13) $$\begin{array}{l} P_{\text{pre}}=\frac{N_{\text{TP}}}{N_{\text{TP}}+N_{\text{FP}}} \end{array}$$

(14) $$\begin{array}{l} P_{\text{rec}}=\frac{N_{\text{TP}}}{N_{\text{TP}}+N_{\text{FN}}} \end{array}$$

(15) $$\begin{array}{l} P_{\text{F1}}=2\cdot \frac{P_{\text{pre}}\cdot P_{\text{rec}}}{P_{\text{pre}}+P_{\text{rec}}} \end{array}$$

(16) $$\begin{array}{l} P_{\text{acc}}=\frac{N_{\text{TP}}+N_{\text{TN}}}{N_{\text{TP}}+N_{\text{TN}}+N_{\text{FP}}+N_{\text{FN}}} \end{array}$$

**Figure 7:**
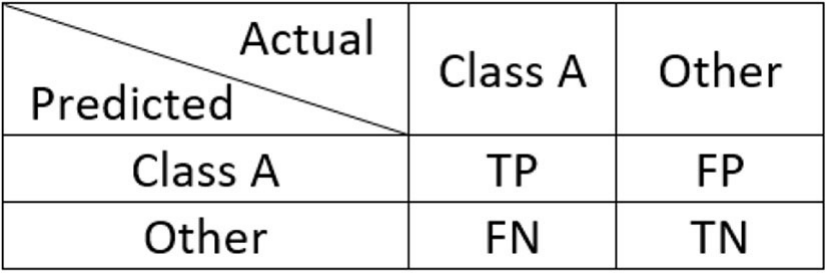
Classification of TP, TN, FP, and FN samples.

Here, *N*_TP_, *N*_TN_, *N*_FP_, and *N*_FN_ represent number of samples that are true positive, true negative, false positive, and false negative, respectively, as illustrated in Figure 7. *P*_pre_, *P*_rec_, *P*_F1_, and *P*_acc_ are the performance measures that represents precision, recall, F1-score, and accuracy, respectively. Of these measures, precision, recall, and F1-score are defined in the range of 0–1, whereas, accuracy is expressed in percentage.

Using these four parameters we can evaluate the classification performance comprehensively and in an unbiased manner. From mathematical representations, we can see that the fundamental difference between accuracy and other parameters is TN samples. In our designed experiment the number of samples in each class is not consistent. In such cases precision and recall can also provide very useful insight into classification performance. Taking the example of rest task, precision calculates from the total number of samples that are classified as rest how many were actually rest. Meanwhile, recall calculates, from the number of times a user was instructed to keep rest state, how many samples were correctly identified as rest state. Finally, the F1-score tells the balance between precision and recall.

## 4. Experiments and Results

With the developed method, three experiments are performed, i.e., task identification, influence of sensor placement, and grasping assistance. Details and results of each task are provided in forthcoming sections.

### 4.1. Task Identification

Six subjects participated in this experiment. All of them were healthy, right-handed, and aged between 25 and 35 years. Ethical approval for these experiments was obtained from an ethical committee, Region Nordjylland, Denmark.

**Figure 8:**
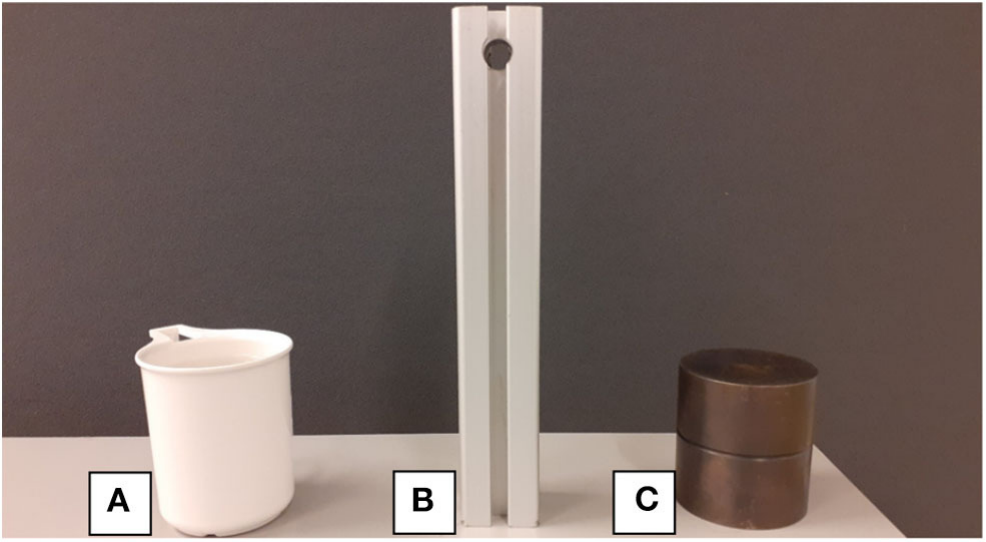
Objects of different shape and weight that are grasped during task identification experiment, **(A)** empty cup, **(B)** aluminum bar, and **(C)** solid metal cylinder.

In this experiment, performance measures, i.e., precision, recall, F1-score, and accuracy, are computed to evaluate the classification performance. For this purpose, an experiment was designed where a subject performs hand opening and closing, first without any object and afterwards with three objects, as shown in Figure 8, of different attributes.

The protocol of the experiment is as follows: the subject is instructed to sit in a chair with their hands resting on the table beside the objects. The first task the subject performs is calibration, as explained in section 3.1, which is followed by a rest state gesture, as shown in Figure 4C, which is held for 5 s to determine the threshold limits. Afterwards, real-time testing tasks are performed in which, for open and close tasks, the subject lifts his/her hand from the table and keeps it in open state, as shown in Figure 4A. The subject closes his/her hand when the instruction is shown on the screen and opens it up when the instruction to open is shown on the screen again. The subject is instructed that an open hand posture should be maintained throughout the experiment. For the grasp task, hand is lifted from the table and kept open, as shown in Figure 4A. When the grasp instruction is shown on the screen, subject grasps the object and slightly lifts it from the table with a small clearance of approximately 1.0 cm.

**Figure 9:**
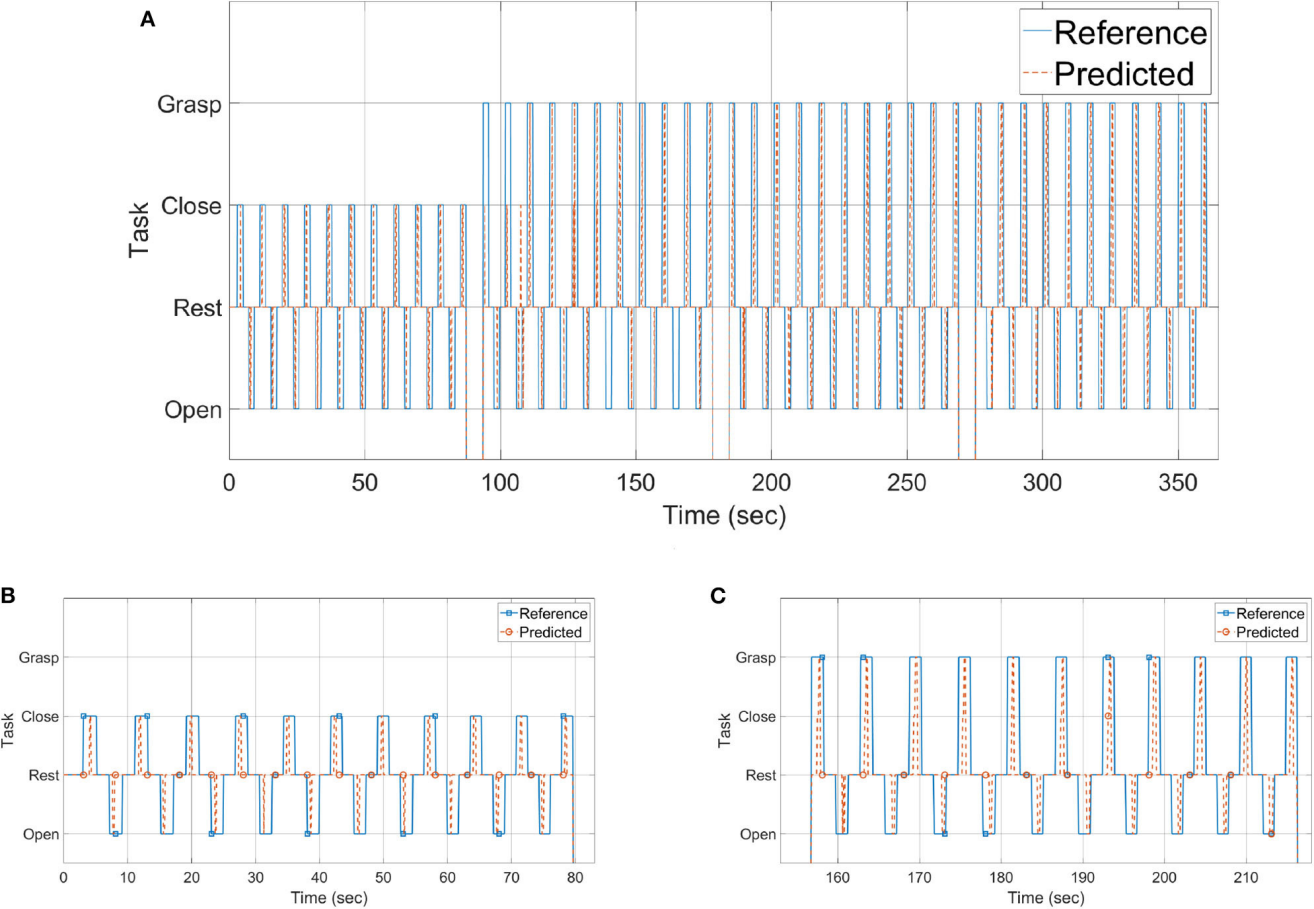
Tasks performed during **(A)** the whole span of time, **(B)** opening and closing of the hand, and **(C)** grasping object B.

**Figure 10:**
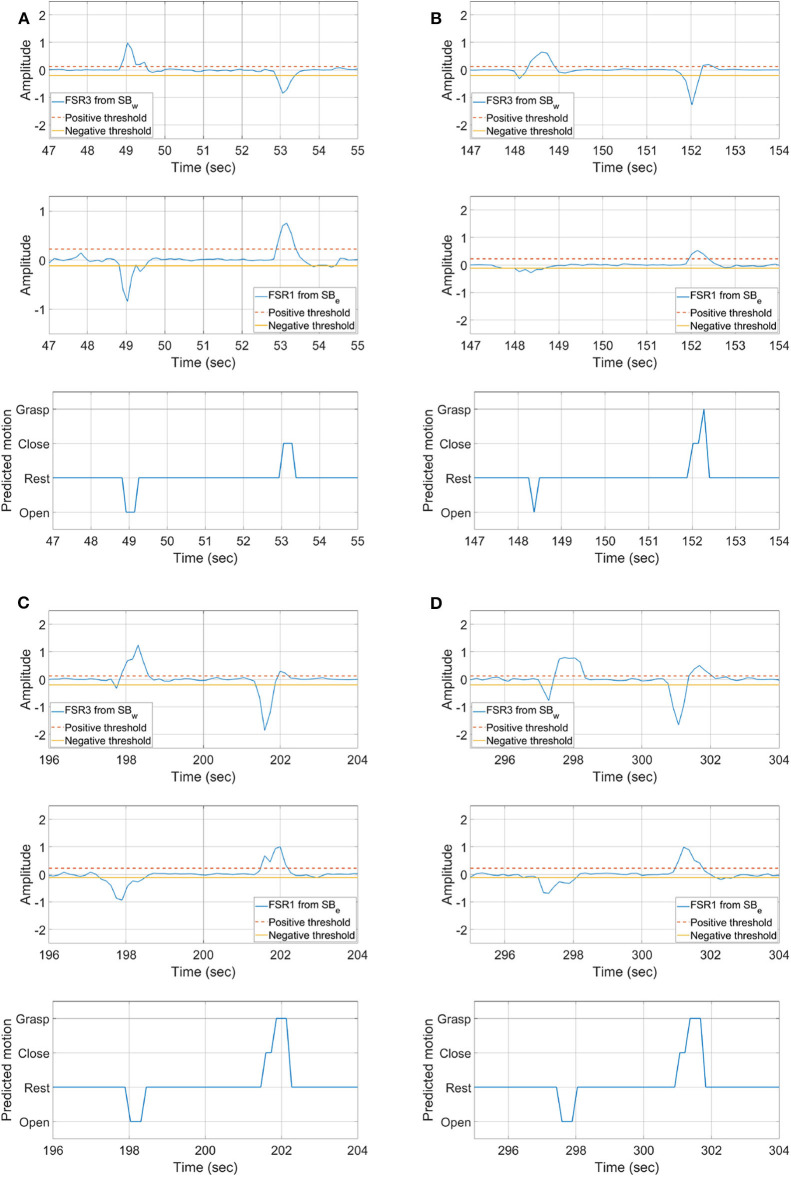
Results of single instances **(A)** open/close, grasping objects **(B)** A, **(C)** B, and **(D)** C, shown in Figure 8.

**Figure 11:**
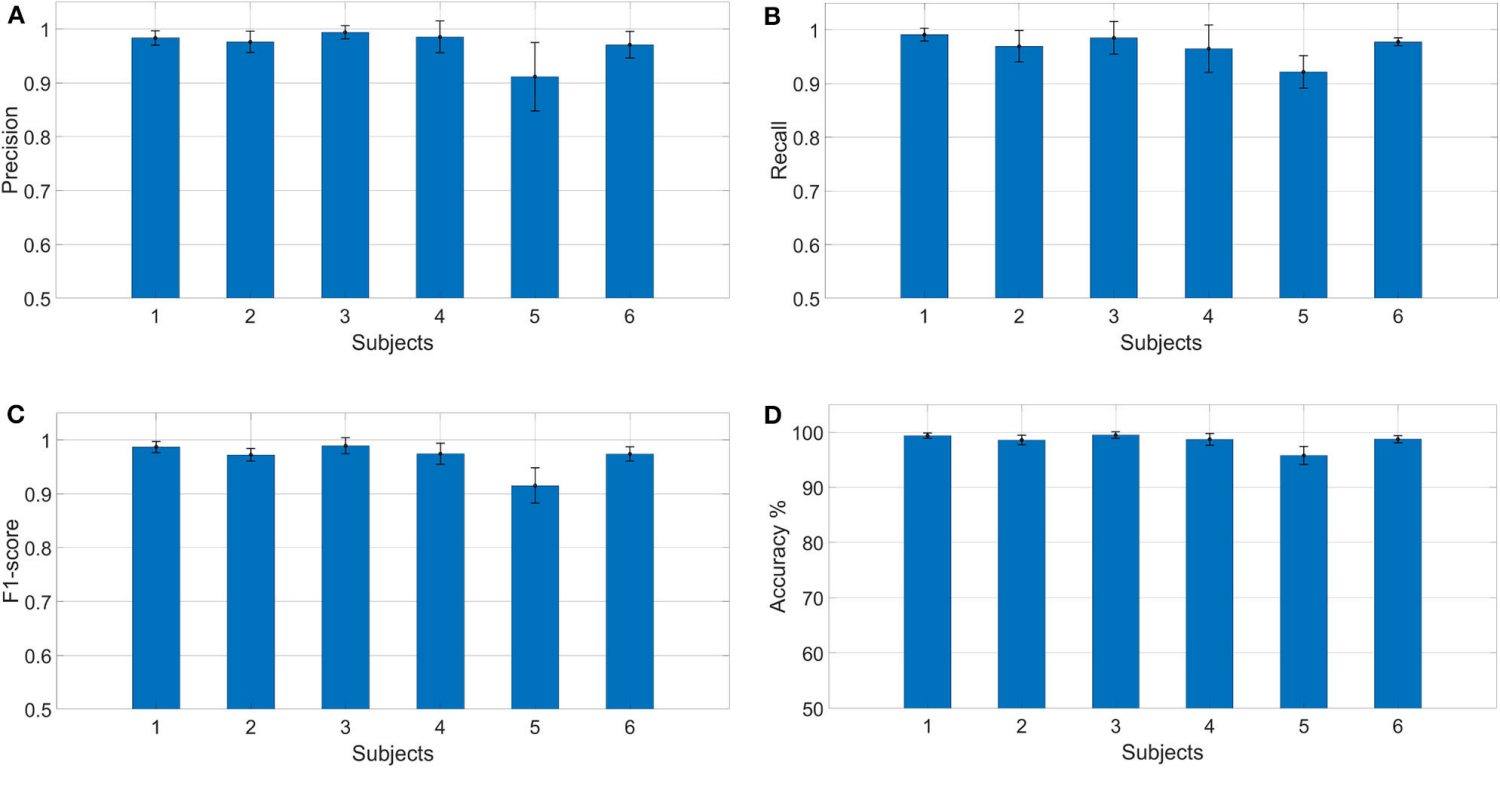
Results calculated for each subject individually **(A)** precision, **(B)** recall, **(C)** F1-score, and **(D)** accuracy.

**Figure 12:**
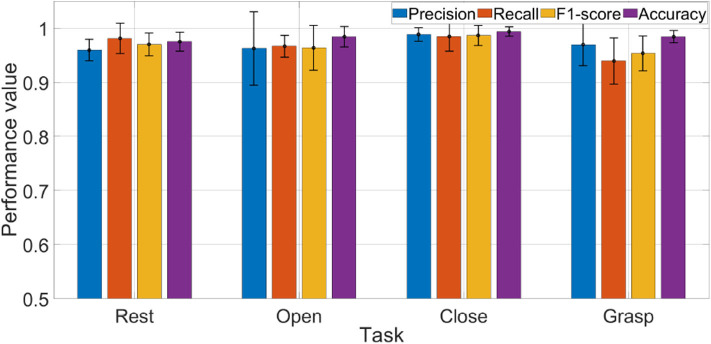
Average results of each performance measure w.r.t each task. Accuracy plot is shown normalized between 0 and 1.

The results of the experiment are shown in Figures 9–12 and summarized in Table 2. Figure 9 shows the experimental results for one of the subjects. Figure 9A shows the reference and predicted tasks. In the first 80 s of the experiment, the subject is instructed to perform the rest, open, and close tasks. From *t* = 80 to 155, *t* = 155 to 220, and *t* = 220 to 285 s, the subject is instructed to grasp objects A, B, and C sequentially. In this figure, the solid blue line shows the task to be performed and the dotted red line the result predicted by a classifier when a subject performs that particular task. A zoomed-in view of open and close tasks is shown in Figure 9B and of grasping task for object B is shown in Figure 9C.

**Table 2:** Average results of performance measures calculated for each subject.

**Performance measures**	**Precision**	**Recall**	**F1-score**	**Accuracy %**
Subject 1	0.98 ± 0.013	0.99 ± 0.012	0.99 ± 0.010	99 ± 0.5
Subject 2	0.98 ± 0.019	0.97 ± 0.029	0.97 ± 0.011	99 ± 0.9
Subject 3	0.99 ± 0.012	0.98 ± 0.030	0.99 ± 0.014	99 ± 0.6
Subject 4	0.99 ± 0.029	0.96 ± 0.044	0.97 ± 0.019	99 ± 1.0
Subject 5	0.91 ± 0.063	0.92 ± 0.030	0.92 ± 0.032	96 ± 1.6
Subject 6	0.97 ± 0.024	0.98 ± 0.007	0.97 ± 0.013	99 ± 0.7
Average	0.97 ± 0.029	0.97 ± 0.024	0.97 ± 0.027	98 ± 1.3

Single instances of abovementioned tasks are shown in Figure 10. Figure 10A is the result of an open and close task. The results show that, initially, the hand was in the close state; as the subject opens the hand, a drop in signal amplitude near the elbow and an increase in signal amplitude near the wrist joint is observed. The classifier is able to detect that the hand is opened as the movement is performed. Afterwards, when the hand is closed, the inverse muscle activity pattern can be seen, and, as the movement is performed, the classifier is again able to detect that the hand is closed.

The instances of grasping object A, B, and C are shown in Figures 10B–D, respectively. Data is presented in the same order as represented for Figure 10A. Initially, the subject is holding the object. As the hand is opened, it is seen from the FSR readings that their associated muscle contraction near the wrist increases, and contraction near elbow is decreased. From the opened hand state when the subject is instructed to grasp the object, it can be seen that classifier first detects that the hand is closing. It can also be seen from the FSR readings that it is increasing near the elbow and decreasing near the wrist, indicating hand closing. As the object is grasped, an increase in readings on both sensor bands is seen, and the classifier correctly detects that an object is being grasped. These results show that the threshold-based classifier is able to distinguish between all four motion states, i.e., rest/steady, open, close, and grasp, accurately.

Results in terms of precision, recall, F1-score, and accuracy are shown in Figures 11, 12 and Table 2. In the figures, the error bar represents the performance deviation within the tasks, i.e., rest, open, close, and grasp.

The average performance values w.r.t each task are shown in Figure 12. Considering the rest state, it can be seen that average recall value is 0.98, which reveals that only 2% of the rest states were not detected. It is to be noted that rest state was held in all postures, i.e., open hand, close hand, and grasp. In the context of real-time operation, this result is very critical. Any miss-classification can cause undesirable movement/action, especially if subject is holding an object. The results show that the algorithm is highly accurate in detecting the rest state. Precision for detecting rest state is equal to 0.96, which shows that in only few cases where subject was performing another task (open, close, or grasp), classifier detected it as rest state.

For open and close tasks, it can be seen that recall and precision scores are very similar. For grasp, we can see that precision (0.97) is higher than recall (0.94). From precision, we can deduce that, of all the tasks that were classified as grasp, only 3% of them were miss-classifications. Meanwhile, the recall result tells us that 6% of the times when a subject grasped an object, the classifier did not detect it as grasp. To improve precision, the threshold level should be raised, but this will affect the performance of recall. Raising the threshold will have the opposite impact on other performances. It will improve the recall but might reduce the precision. With the current setup, classification performance of the algorithm depends on the trade-off between recall and precision. Depending on the applications, threshold levels can be tuned to get better results. The performance can be improved by incorporating more FSR sensors or by using more features for threshold determination.

### 4.2. Influence of Sensor Placement

**Figure 13:**
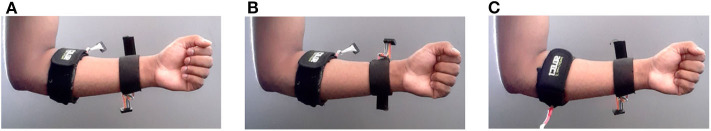
Three placements of sensor bands, **(A)** two FSR1 from SBe and SBw are aligned with brachioradialis and near insertion of brachioradialis, **(B)** aligned with brachioradialis and flexor carpi ulnaris, **(C)** aligned with palmaris longus and near insertion of brachioradialis.

In this experiment, the effect of sensor placement on motion detection is studied. To achieve this objective sensor bands are placed over the forearm in three different orientations/placements, as shown in Figure 13. In placement A, FSR1 from sensor bands *SB*_*e*_ and *SB*_*w*_ is aligned with brachioradialis and near insertion of brachioradialis. In placement B, it is aligned with brachioradialis and flexor carpi ulnaris muscles. Finally, in placement C, it is aligned with palmaris longus and near the insertion of brachioradialis.

Tasks performed for each placement of sensor bands are as follows:

- Open and close of hand without grasping any object
- Grasping object C as shown in Figure 8.

**Figure 14:**
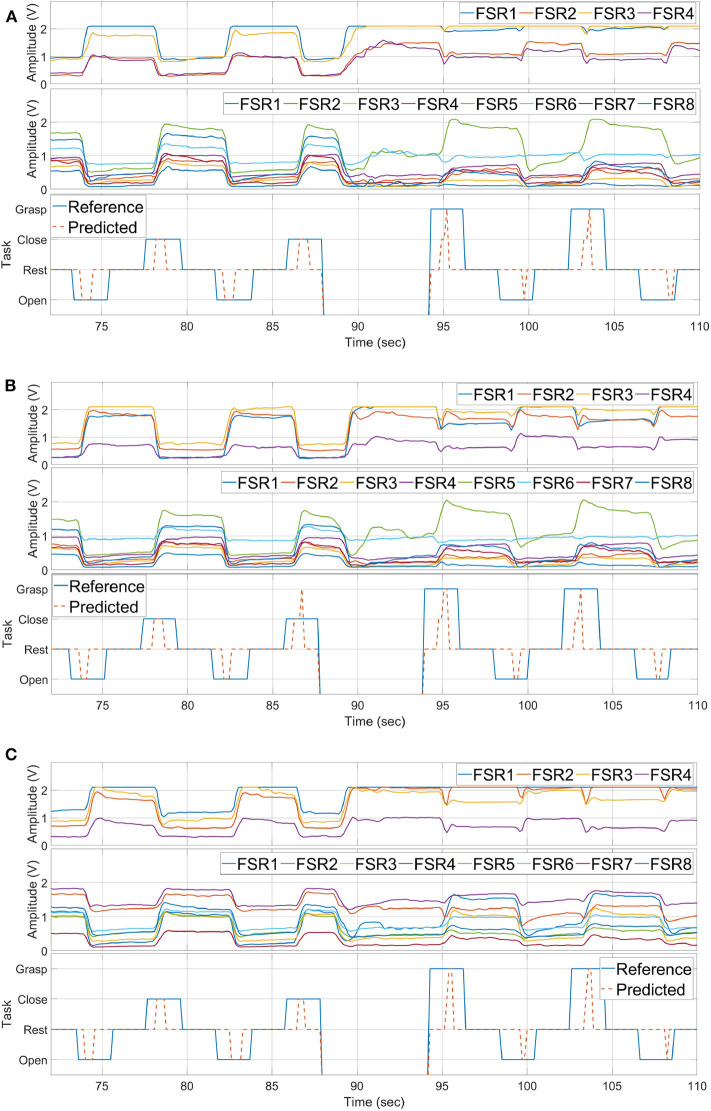
Hand motion detection with three placements of the sensor bands, **(A)** with placement A, **(B)** with placement B, **(C)** with placement C.

Each task is performed 10 times under same conditions as explained in section 4.1. The results of each experiment are shown in Figure 14, where Figures 14A–C are the results of placement A, B, and C, respectively, by sensor band orientation. In each sub-figure of Figure 14, the first figure is the FSR sensors data from the sensor band placed near the wrist, and the second is the data of FSR sensors placed near the elbow, and the third figure displays the reference and predicted tasks.

Even though the raw data is not similar for each sensor placement, the developed method is able to detect all four hand gestures accurately. The performance of task detection is less affected. As seen from predicted results, rest state, hand opening, closing, and grasping achieved the average accuracies of 98.15, 99.24, 100, and 98.16% for all three placements.

### 4.3. Grasping Assistance

**Figure 15:**
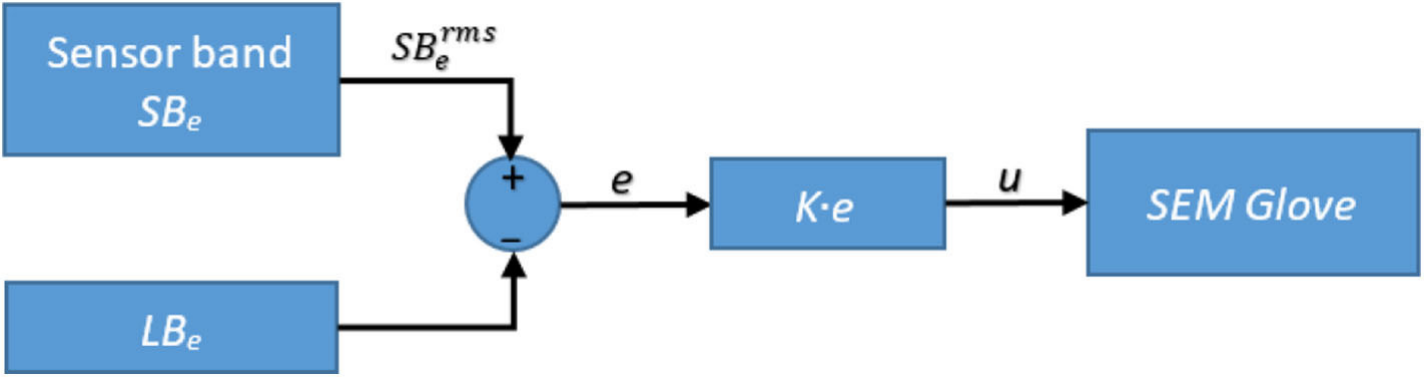
Block diagram of the exoskeleton control.

In this work, grasping assistance is provided using SEM Glove where the desired assistance level is regulated by implementing a proportional control scheme. The block diagram of the control scheme is shown in Figure 15. Referring to Equation (11), the input of the proportional control is the average MCI force measured by the sensor band placed near the elbow, and the output *u* is then relayed to the exoskeleton. Moreover, grasping assistance provided by SEM Glove is further validated by measuring the grasping force through force sensors embedded inside SEM Glove exoskeleton.

**Figure 16:**
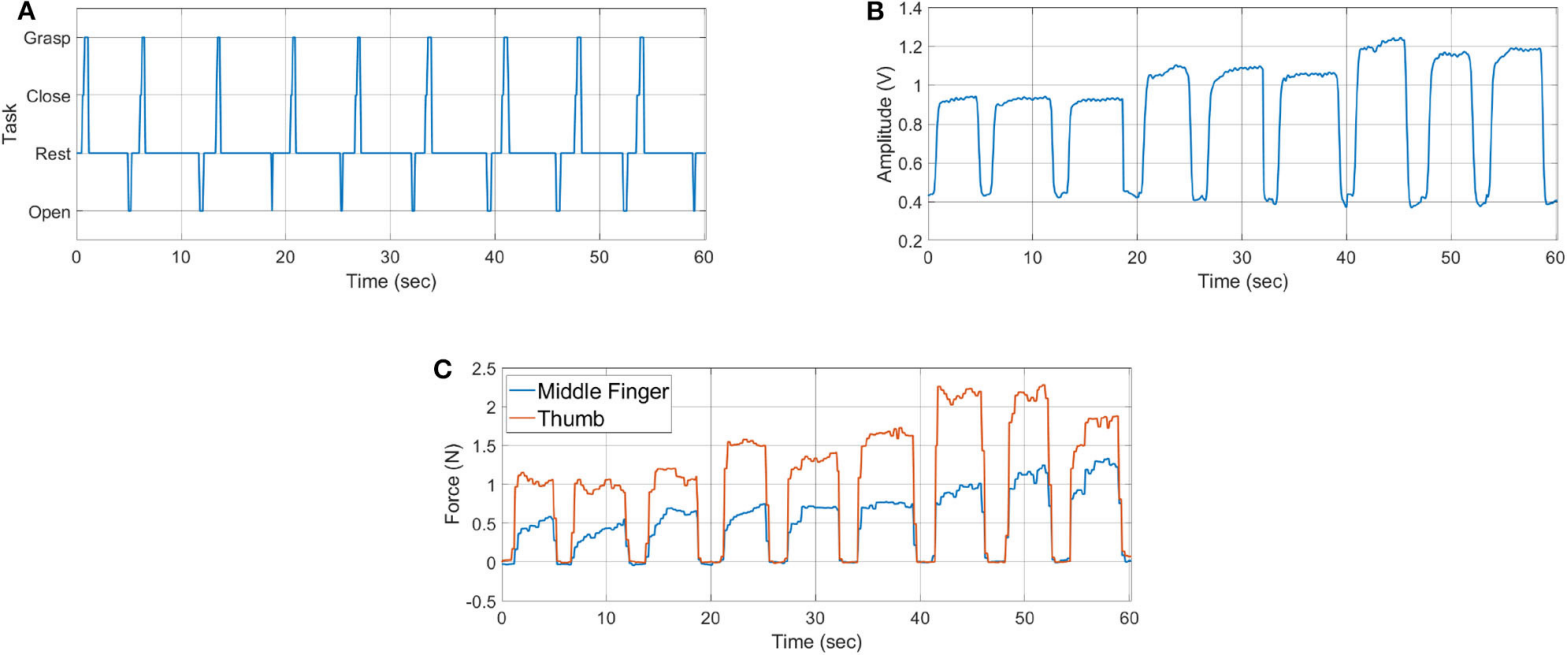
Hand exoskeleton control results: **(A)** task identified, **(B)** MCI force measured from sensor band placed near elbow joint, and **(C)** assistance force provided by SEM Glove.

In this experiment the sensor bands are worn on right forearm and exoskeleton is worn on the left hand. Furthermore, three different payloads, i.e., 1.2, 2.3, and 3.4 kg, applied from *t* = 0 to 20, *t* = 20 to 40, and *t* = 40 to 60 s, respectively, are being grasped for three times each. The results of the experiment are shown in Figure 16.

**Figure 17:**
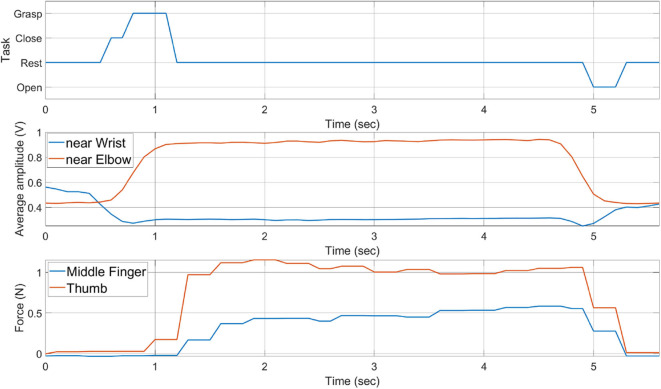
History of task performed, average MCI forces, and grasping forces measured by SEM Glove.

Figure 16A shows the task predicted by the classifier. Net MCI force measured by the *SB*_*e*_ sensor band is shown in Figure 16B. The resulting grasping force measured from SEM Glove sensors is shown in Figure 16C. Whereas, the single instance of grasp task is shown in Figure 17. With the detection of a grasping task and MCI force, assistance is provided by the exoskeleton, which is evident from the sensor reading of the SEM Glove.

If we look closely at Figures 16B,C, we can see that the MCI forces are increasing with the payload grasped by the subject. It is also seen that the forces read by the sensors placed at the middle finger and thumb are increasing with the payload. These are the grasping forces that are caused by the physical interaction between fingertips and the object. When assistance provided by the exoskeleton is increased, the exoskeleton will help to grasp the object tightly and in turn grasping force measured the sensors, placed in finger tips, will increase. This validates that with the increase in MCI force, shown in Figure 16B, exoskeleton is able to provide the grasping assistance accordingly.

## 5. Discussion

In this work a novel method is developed for hand motion detection and for the provision of assistance in carrying out an object grasping task. We also addressed the challenge of data collection for training and proposed an alternative solution for it.

The new method is advantageous in reducing the complexity and increasing the usability of the system for a longer period. In an AI-based pattern recognition method, obtaining a correct and sufficient training dataset is one of the major challenges. Moreover, even if the training data is obtained correctly there still exists another challenge of reusing it from time to time. The reason is due to the placement of sensor at the exact location and change in muscle activity levels. The method proposed in this work effectively addresses these challenges. The method requires sensor calibration and rest state data of the hand. Afterwards, the system can detect the hand motions based on change in activity level. Additionally, the requirement on placing sensor band at exact location is mitigated. Moreover, the calibration procedure increases the sensor's sensitivity and solves the problem of sensor resolution if the band tightness is changed from one day to the next.

Another advantage of this method is the dual working modes of the sensor band. Besides motion recognition, the sensor band is also used to control assistance level in grasping an object, which is proportional to the MCI force measured.

The results in this work are significant for physical assistance in workplaces. For a workplace environment, it is critical for any solution that it be accurate, robust, involving less training, and is not sensitive to environmental conditions. With these requirements in mind, comparing our method to other detection methods like sEMG, which is highly prone to noise that is caused by sensor placement, orientation, and skin conditions, our method is less affected by skin condition and can be worn without very exact orientation and placement. Moreover, our developed method has the advantage of using small training datasets. In Arteaga et al. (2020) and Pinzón-Arenas et al. (2019), each gesture was repeated for more than 10 times. Whereas, in our method beside calibration, rest data is recorded for only one time. By this advantage the user can take off the device and put it back on conveniently without worrying about its performance.

This novel method using FSR sensor bands offers a robust and accurate alternative for human-robot interaction. The works presented in this paper and in previous studies (Islam et al., 2018; Islam and Bai, 2019) have shown that FSR-based sensor bands can be applied for control of upper-body assistive exoskeletons in different ways. Beside these, sensor bands can be applied for other types of applications of upper-limb and lower-limb exoskeletons. Moreover, this method can be used to assess the muscle activities for medical purposes and design of control strategies.

Besides these advantages, some limitations of the method are noted. External contact with the sensor band can change the sensor readings, which can result in incorrect motion detection. Hand motion speed is also a factor that can lead to miss-classification. If the motion is performed at slow speed, the algorithm might not be able to detect the task. These challenges can be addressed by either placing the FSR array outside of the sensor band or by implementing robust AI techniques for fault detection. Movement speed challenge can be addressed by increasing the window size during features extraction stage. However, increasing the window size can introduce delay in exoskeleton response.

## 6. Conclusions

This work is aimed at developing an effective and convenient method to detect hand motions, i.e., rest, open, close, and grasp, using FSR-based sensor bands, which is further used to control hand exoskeleton and provide assistance in grasping task. The objectives are achieved by developing a threshold-based task detection algorithm to determine the hand motion, which is based on the change in MCI forces. Moreover, with the detection of grasping task a proportional force control is also implemented to provide assistance through a soft hand exoskeleton.

The contribution of this work is to experimentally validate whether the sensor bands can be used to detect hand motion and to implement proportional assistance control. Detection of hand motion with the requirement of minimal training data and its validation with testing on multiple subjects are other contributions of this work. The results showed that the developed method can detect each task with high precision, recall, and accuracy. Furthermore, experimental verification of proportional assistance control with SEM Glove in a grasping task is another contribution of this work. The results have shown that the developed method can be used with soft exoskeleton to assist workers in grasping tasks.

In this work, experiments were performed in a controlled environment. In order to test the method for daily routine activities, our future work will focus on sensor fusion techniques to improve robustness against disturbances, which can be caused by other limb movements. Furthermore, the method can be extended to detect other hand gestures and elbow and lower extremity motions.

## Data Availability Statement

The raw data supporting the conclusions will be made available by the lead author on reasonable request.

## Ethics Statement

The studies involving human participants were reviewed and approved by ethical committee, Region Nordjylland, Denmark. The participants provided their written informed consent to participate in this study.

## Author Contributions

MI and SB defined and developed this research work. MI developed the initial protocol draft, collected data, performed the analysis, and wrote the first draft of the manuscript. SB finalized the protocol, reviewed the manuscript, and approved the final version. Both authors contributed to the article and approved the submitted version.

## Conflict of Interest

The authors declare that the research was conducted in the absence of any commercial or financial relationships that could be construed as a potential conflict of interest.
